# The Urgent Need for Precision Medicine in Cancer and Its Microenvironment: The Paradigmatic Case of Multiple Myeloma

**DOI:** 10.3390/jcm11185461

**Published:** 2022-09-16

**Authors:** Antonio Giovanni Solimando, Markus Krebs, Max Bittrich, Hermann Einsele

**Affiliations:** 1 Guido Baccelli Unit of Internal Medicine, Department of Biomedical Sciences and Human Oncology, School of Medicine, Aldo Moro University of Bari, 70124 Bari, Italy; 2 Department of Urology and Pediatric Urology, University Hospital Würzburg, 97080 Würzburg, Germany; 3 Comprehensive Cancer Center Mainfranken, University Hospital Würzburg, 97080 Würzburg, Germany; 4 Department of Internal Medicine II, University Hospital Würzburg, Josef-Schneider Straße 2, 97080 Würzburg, Germany

Precision medicine is particularly relevant for cancer and microenvironment deconvolution for therapeutic purposes in hematological and non-hematological malignancies [1,2,3,4]. Multiple myeloma (MM) is no exception, representing a paradigmatic condition. Indeed, although the advent of next-generation sequencing uncovered the genomic makeup, MM is a disease characterized by high genetic complexity [5,6]. Primary events, including IgH translocations and hyperdiploidy, and secondary events such as CNV, SNP structural variations, and epigenetic changes also occur [7,8,9]. Intra-patient/intratumoral and interpatient heterogeneity, given the subclonal population’s extreme dynamics, starts from the beginning and the asymptomatic condition through all the stages of the disease [10,11,12]. On top of this, treatment selection further boosts the disease complexity, while prompting novel therapeutic strategies [13,14]. Treatment options are also heterogeneous [15]. The large number of therapeutic alternatives currently available for MM patients gives rise to a wide range of possible clinical settings that cannot be easily covered by a single algorithm [16].

Multimodality spans clinical, molecular, and imaging levels. Several factors such as demographics, biochemical properties, staging, tumor burden (BMPC), minimal measurable disease, transplant status, treatments, and outcomes should be combined with cytogenetics, RNAseq, microarray, and novel tools (such as scRNASeq, WES, WGS, targeted sequencing, methylation, and proteomics). Imaging techniques, such as PET-CT scans and MRIs are also relevant. This landscape represents a typical framework for a machine learning approach [5]. The main issue of the existing valuable datasets in the literature is that they are very sparse regarding the specific modalities [17]. The attempts made to define simple features with high predictive power have been validated in the investigations regarding the progression from MGUS/SMM to MM or the survival risk [18,19,20]. Despite being effective, these simple, unimodal models lack effectiveness in some patient subgroups, namely the ultra-high-risk patients [21]. Thus, crowdsourced efforts have been made to develop machine learning models of rapid clinical progression by 18 months in newly diagnosed MM patients using a combination of DNA-, RNA-, age, ISS, and other demographic, clinical, or cytogenetic-based features [22]. Mason et al. benchmarked their machine learning approach against previously published models. In this DREAM challenge study, the data-driven risk predictive models leveraged high-dimensional, multimodal, and multiexperimental data, and the analysis of top-performing methods identified the high expression of PHF19 as the gene with the strongest association with MM progression [22]. Although an attempt to address patient subgroup stratification has also been made [17], validation is largely needed in real life. Collectively, the challenges for precision medicine in MM are constituted by the heterogeneity of the disease, also considering the treatments available in clinical practice. Mostly, the existing datasets are “small”, soiled, and sparse, and multimodal resources are still lacking. Therefore, multimodal and federated approaches to breaking data silos are mainly needed [23].

An integrative modeling approach might represent a possible integration of the data and prior biomedical knowledge to overcome data limitations [24,25]. This approach would imply a shift from a gene-centric to a network approach. Multiple myeloma represents a landscape in which more precise identification of high-risk disease relapse has been possible based on these novel approaches [26]. Based on the transcriptome, Wall et al. developed a complex pipeline eventually able to associate a more efficient signature with each subgroup, in terms of network activity rather than gene activity, with the advantage of being more interpretable [26].

Similar approaches have also been applied to more complicated projects, namely a personalized assessment of the risk of progression from MGUS/SMM to MM [27]. These studies highlight that even though simple predictors (such as age) perform well, the specific landscape of the genomic makeup characterizes stable and progressive subgroups [27]. One major challenge is related to the lack of access to a high volume of content data in the pre-malignant phases.

There is an urgent need for precision medicine in cancer and multiple myeloma [28]. The sparsity of datasets in the literature represents significant constraints on crucial modalities on single datasets compared with the disease’s level of heterogeneity. Machine learning models have already been proven valuable as complementary tools to well-established clinical and bioinformatic approaches. By leveraging multimodalities and prior biomedical knowledge, artificial intelligence can provide a more efficient risk score and suggest new biomarkers and subgroup–treatment associations [22,29,30].

Overall, we are confident that the issues raised in this editorial might help to interpret the findings of the current clinical and pre-clinical practice. The authors plan to generate questions about the optimal treatment approach for cancer therapy and its microenvironment, considering the novel tools presented.
